# Towards the Functional Ageing of Electrically Conductive and Sensing Textiles: A Review

**DOI:** 10.3390/s21175944

**Published:** 2021-09-04

**Authors:** Christian Biermaier, Thomas Bechtold, Tung Pham

**Affiliations:** Research Institute of Textile Chemistry and Textile Physics, Faculty of Chemistry and Pharmacy, University of Innsbruck, 6850 Dornbirn, Austria; Christian.Biermaier@uibk.ac.at (C.B.); Thomas.Bechtold@uibk.ac.at (T.B.)

**Keywords:** e-textiles, conductive textiles, functional ageing, durability, endurance, fatigue lifetime, textile sensors, flexible sensors, accelerated ageing

## Abstract

Electronic textiles (e-textiles) have become more and more important in daily life and attracted increased attention of the scientific community over the last decade. This interdisciplinary field of interest ranges from material science, over chemistry, physics, electrical engineering, information technology to textile design. Numerous applications can already be found in sports, safety, healthcare, etc. Throughout the life of service, e-textiles undergo several exposures, e.g., mechanical stress, chemical corrosion, etc., that cause aging and functional losses in the materials. The review provides a broad and critical overview on the functional ageing of electronic textiles on different levels from fibres to fabrics. The main objective is to review possible aging mechanisms and elaborate the effect of aging on (electrical) performances of e-textiles. The review also provides an overview on different laboratory methods for the investigation on accelerated functional ageing. Finally, we try to build a model of cumulative fatigue damage theory for modelling the change of e-textile properties in their lifetime.

## 1. Introduction

Electronic textiles or e-textiles become more and more important in daily life and attract increased attention of the scientific community over the last decade. This interdisciplinary field of interest ranges from material science, over chemistry, physics, electrical engineering, information technology to textile design. Typically, e-textiles are defined as fabrics or cloths having sensors, actuators, power sources and data processors as integrated parts. All these parts are electrically connected by conductive lines [1].

Often, e-textiles are associated with the term “smart textiles”. According to common understanding, smart textiles are, however, defined as “materials that sense and respond to environmental stimuli” [2,3]. The term “environmental stimuli” can be understood as chemical, electrical, thermal, mechanical, or other exposures. Smart textiles can also be divided into “passive smart textiles”, e.g., humidity, temperature, pressure, gas sensors and “active smart textiles” which adapt their behaviour to their environmental exposure [3]. Apart from the typical functionality as a connector for signal transport and energy supply, conductive structures can also be utilised as a sensor and actuator areas in modern smart textiles, e.g., as temperature sensors, [4] pressure sensors [5] and piezo elements for energy harvesting [6].

In any case, electrical conductivity is an essential part of e-textiles, which is the focus of the review. In recent decades, many techniques have been developed to introduce conductive lines and patterns into textiles [7]. As the most important success criterion, the conductivity is often reported. However, the evolution of the functionality over usage time—where worsening is observed in most cases—is not yet well understood. Beside chronological age, the term ageing is used as an abstract term for a change on the material properties during usage or storage.

The classical ageing of textile can be categorised into physical ageing, photochemical degradation, thermal degradation, chemical attack and mechanical stress. Physical ageing is recognised as the re-crystallisation of the semi-crystalline polymers of the textile during usage, leading to a higher crystallinity degree and higher rigidity. Photochemical degradation results from light exposure and leads to the breakage of chemical bonds and possibly to the formation of new bonds. Thermal degradation is, depending on the temperature and exposure time, a kind of physical ageing, or a change in the chemical structure of the polymer considering oxidation and decomposition. Chemical attacks can be observed by an acidic or basic environment, depending on the polymer structure. Mechanical ageing, also referred to as mechanical stress, which results from movement of the textile, is divided into instantaneous response from elastic behaviour and a delayed response due to viscous behaviour [8].

With respect to e-textiles, the ageing phenomena consist of both substrate ageing and the ageing of electrical functionalities. While substrate ageing refers to changes in the material performance, e.g., mechanical strength, chemical inertness, etc., of the fabrics or fibres, functional ageing concerns losses in sensor behaviour, conductivity, capacitance, etc. It is worth noticing that both ageing mechanisms are strongly connected to each other. As an example, worsening in flexibility of a fabric as a result of fibre polymer degradation due to UV irradiation can directly impact the electrical property of the coated conductive paste under bending/stretching conditions. Therefore, it is not always possible to investigate the two ageing processes separately.

As e-textiles increasingly find applications in sport, medical and safety areas, there is a pressing need for ensuring and improving the reliability and durability of the products, in particular when it comes to sensitive applications such as in healthcare and life-saving. Thus, scientific knowledge in ageing behaviour is crucial.

To provide the readers with an overview on different basic mechanisms of functional ageing of e-textiles, in this review, we limit our focus to the changes and damages of conductive functionality as a basic feature of e-textiles under usage conditions and exclude the discussion on the ageing of other electronic devices. Furthermore, different approaches in modelling of functional ageing are discussed.

## 2. Conductive Textiles

In order to better understand the mechanism of functional ageing, especially the electrical conductivity of e-textiles, this section provides an overview of the basic possibilities in integrating electrical conductivity into fibre and textile structures as the integration technology also impacts the functional ageing of e-textiles.

Common techniques to obtain conductivity on yarns or textiles are, for example, metal deposition [9], embroidery of metal yarns into fabrics [10] and the application of metal-containing pastes and inks [11]. These conductive pastes and inks consist of a conductive filler distributed in a printable polymeric matrix. Often, also graphite and carbon black are used in conductive inks [12,13] as well as graphene [14] or types of carbon nanotubes (CNT) [15].

Another concept is the usage of intrinsically conductive polymers (ICP), discovered by A. J. Heeger, MacDiarmid and Shirakawa, achieving the Nobel Prize in 2000. Typically, conductive polymers consist of two main constituents: an extended π-electron system and a doping agent, e.g., an acid for protonation in polyaniline (PANI). Chemical doping by charge transfer or electrochemical doping are also possible [16]. Among a variety of conductive polymers, mainly polyethylenedioxythiophene (PEDOT:PSS) [17], polypyrrol (PPy) [18] and polyaniline (PANI) playing a role for conductive textiles [19].

The conductive material can be applied to a fibre or yarn for later integration into fabrics or the formation of conductive structures can be realised directly on fabric surfaces.

### 2.1. Conductive Yarns

There are many ways to introduce conductivity into textile yarns. One of the most common techniques is the utilisation of conductive polymers or carbon derivatives as conductive material in yarns.

The introduction of conductivity can be implemented in the melt mixing process. Here, the conductive polymer along with additives is mixed with thermoplastic polymers such as polyolefins and melt spun into conductive fibres. Typical examples are shown by Ikkalaa et al. and Yang et al. for PANI in combination with LDPE. This method is simple and the main advantage is the good distribution of conductive material across the entire fibre cross section [20,21]. Another example is compounding a small amount (7%) of multi-walled carbon nanotubes (MWCNTs) in polyamide 6 and for spinning conductive PA 6 fibres [22].

To maintain the bulk properties of fibres, conductive polymers can also be applied by coating on the surface of fibres or yarns. An example is given for the deposition of PANI on Nomex fibres by immersing the fibres into a solution and triggering an active polymerisation reaction [23]. The polymerisation of a conductive polymer may also take place on the surface of impregnated threads [24]. Furthermore, dip coating of fibres into a dissolved conductive polymer, such as commercially available polyaniline emeraldine salt in xylole, can also be applied [22]. The coating of yarns and fibres with carbon derivatives is also available in many varieties. For example, a bovine serum albumin pre-treated polyamide 6 yarn was coated with graphene oxide (GO) and consecutively reduced (i.e., RGO formed) with hydrogen iodide (HI) [14]. Additionally, the pre-impregnation of fibres with polymers (e.g., PVA) can be utilised to enhance the adsorption of carbon nanotubes (CNTs) [25].

Apart from melt mixing and coating, it is also possible to utilise a solvent spinning process for conductive polymers. A mixture of 2-acrylamido-2-methyl-1-propanesulfonic acid and PANI in dichloro acetic acid can be forced through a spinneret under nitrogen pressure into an acetone coagulation bath [26]. A similar technique was used for PEDOT:PSS dispersion in water that was spun into an acetone bath through a single hole spinneret [27].

Taking silver (Ag) and copper (Cu) as the most efficient conductive materials, the deposition of Ag and Cu on textiles is a fast-growing field. Silver can, for example, be deposited on a substrate by physical vapour deposition [28]. Silver-coated yarns are commercially available in different materials and strengths for the application in e-textiles [29].

The deposition of copper is mainly performed on fabrics. However, there are also some techniques for the coating of yarns with copper, e.g., by electrochemical deposition. This technique requires a conductive pre-coating step, e.g., of carbon nanotubes (CNT) [30].

Besides the electrochemical plating, the electroless copper deposition (ECD) plays an important role in metal deposition. Here, a reducing agent, e.g., formaldehyde, is responsible for the reduction in Cu^2+^ ions to copper metal under basic pH conditions, coming along with the development of hydrogen gas. The reaction is metal catalysed; thus, metal seeding is needed before starting the main deposition reaction. More detailed information can be found in literature reviews on electroless copper deposition [31]. Examples for ECD on yarns are given for copper seeding from copper sulphate with reduction by sodium borohydride on a high performance yarn of poly(p-phenylene-2,6-benzobisoxazole) (PBO) or the use of platinum particles as a catalyst on polypyrrole attached to aramid. Reduction agents here were glyoxylic acid or formaldehyde [32,33].

Another way of using metals as conductor in yarns is to manufacture the yarn from metal fibres, especially stainless steel [34,35] or copper [36] in a plied yarn of filaments or staple fibres, which are commercially available. Furthermore, metal fibres/threads can also be combined with polymers to form ply yarns, known as metal polymer hybrid yarns or metal composite yarns. Stainless steel staple fibre blended with polyester can be used in textile strain sensors [37]. Some recent studies on different blends of stainless steel yarn and viscose or PS show the effect of metal content and number of twists on the yarn conductivity. The higher the metal content, the lower the electrical resistance is. Additionally, the moisture affinity of the polymer fibre influences the resistance of the ply yarn. The fibre with the higher absorption capacity led to about 60% of resistance [38].

The combination of conductive and non-conductive parts in a fibre or yarn can be realised in the form of a core–sheath construction. Here, the two components are distributed spatially as the inner part and an outer layer. Often, the outer layer acts as protection layer of the inner part, e.g., polypropylene-coated conductive yarns [39], metal wires with a twisted carbon nanotube web [40] or a PVDF sheath around a PDOT:PSS core [41].

### 2.2. Integration of Conductive Yarns in Textiles

To manufacture e-textiles, conductive yarns need to be integrated into fabrics. Common methods to integrate a conductive yarn in textiles include knitting and weaving. This means, the conductive yarn becomes a part of the nonconductive yarn network.

A conductive yarn can be integrated into a fabric by weft knitting, which means, to exchange at least one course of nonconductive yarn with a conductive yarn. This formed a strain sensor for an elastomeric lycra fabric with a silver-plated polyamide yarn [42]. The multiple contacts of neighboured yarn loops led to a low resistance in the relaxed state, whereas, during extension, these contacts became lost and the resistance increased. This process is part of the modelling of the resistance of knitted strain sensors by dividing each loop into different micro resistors [43]. The performance of a strain sensor is expressed by the gauge factor (GF), which is defined by ratio of resistance change and elongation. In the shown example, the gauge factor depended on the composition of conductive yarn. Silver-plated yarns show a strong, but steel–polyester hybrid yarns show a weak gauge behaviour [44].

Weaving also provides the possibility of integrating conductive fibres into fabrics. The woven textiles also show a gauge resistance behaviour, which is influenced by the weft density. In an example of a steel–polyester hybrid yarn, woven with a blend of polyester and cotton, the gauge sensitivity decreased with increasing density [45].

Models for the prediction of the resistance behaviour of woven conductive fabrics use micro-resistors and consider the crossings of warp and weft yarns as a critical factor. Models can be reduced to a unit cell of two warp and two weft yarns that consists of 16 different micro resistors [46], working as a textile resistor network [47]. Further models are shown by Beex et al. [48] or Zhang et al. [49].

Apart from the direct integration of conductive threads by knitting and weaving, conductive yarns can also be attached on a textile using embroidery. There are stitching embroidery and tailored fibre placement (TFP) machines [50]. The stitching embroidery is performed by using a conductive yarn as upper or lower yarn and an insulating yarn as counter-part. The flexibility of this method is shown by embroidering different patterns, e.g., spirals that function as electrical inductors or antennas [51].

**Figure 1:**
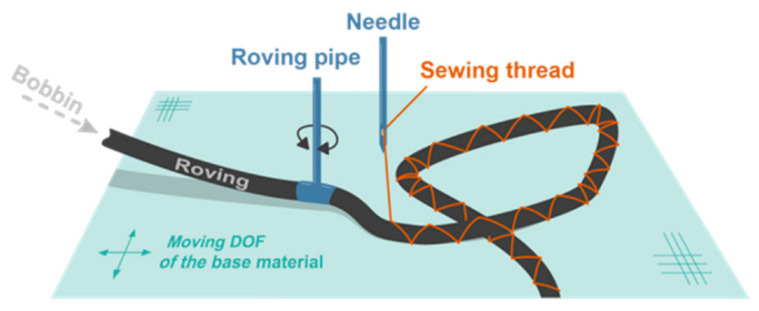
Sketch of tailored fibre placement in technical application [55], reprinted by Creative Commons Attribution (CC BY) license MDPI.

Tailored fibre placement (TFP) (c.f. illustration Figure 1) puts less mechanical stress onto the placed fibre or yarn during its production and is known for placing carbon rovings on textiles. A fibre or bundle of fibres or a yarn is placed on a textile with subsequent zigzag embroidery on top [52]. The manufacturing of different patterns is very easy with TFP. An example is shown for a two dimensional strain sensor manufactured by placing a silver-coated polyamide yarn on a polyester based stretchable fabric [53,54].

### 2.3. Conductive Textiles by Surface Coating and Deposition

Another method of producing conductive structures on fabrics is surface coating with a layer of conductive material. Fabrics are similarly treated as yarns to gain conductivity.

Among others, dip coating is a very simple way of applying conductive materials to fabrics. In an example, a cotton textile was immersed into a colloidal solution of single-walled carbon nanotubes dissolved in 1,2-dichlorobenzene. The conductive textile showed a gauge and piezo-resistive behaviour [56]. Graphene oxide has good adsorption properties on polyethylene terephthalate. The graphene oxide layer needs to be reduced with, e.g., stannous ions after the adsorption to gain conductivity and crosslinking [57]. This technique is also referred to as dyeing [58]. Examples of dip coating that include conductive polymers are the direct application of PEDOT:PSS dispersed in water onto fabrics [59] or in situ synthesis of polythiophene compounds in the presence of a textile substrate [60].

Considered as state of the art, the application of conductive inks and printing conductive lines on flexible substrates have been known since the earlier 1990s. In an example from 1994, a carbon-containing polymer was screen-printed onto a polymer foil for manufacturing a field effect transistor [61]. The panoply of conductive inks includes colloidal solutions and suspensions of conducting materials such as derivatives of carbon or conductive polymers. Solvents are water or organic liquids and contain additives such as a polyethene polyvinylacetate copolymer as a viscosity and stability agent. In an example, a spraying technique was used as a simple way of printing. A textile was sprayed on both sides with conductive ink. As the textile itself was utilised as an insulation layer, a flexible electrical capacitator was formed [62].

A more advanced technique is inkjet printing. Several factors influence the quality of inkjet printing. The surface tension of aqueous solutions is influenced by surfactants and the surface tension of the substrate can be influenced by different activation strategies, e.g., UV radiation, ozone or plasma [63]. An example for a conductive polymer-containing ink is an aqueous dispersion of PEDOT:PSS stabilised with DMSO and ethylene glycol for printing on a PET fabric to deliver the electrical energy for electroluminescence [64].

In general, inks for screen-printing need a higher viscosity than for inkjet printing. Because of the higher viscosity, it is possible to load the ink formulation with a higher conductive solid content. For example, an ink with a content of 60% silver flakes in a vinyl resin was used for screen printing on a textile fabric. The conductive layer was additionally coated with a protective polymer layer on top [65].

Printing methods can also be combined with other deposition techniques. Localised electroless copper deposition becomes possible by pre-printing the metallic catalytic seeds, mainly silver, onto polymeric substrates [66].

Apart from coating and printing, the application of metal deposition techniques obtains more attention with regard to the formation of conductive structures on textile surfaces. It is worth noticing that metal deposition in this context refers to the formation of a metallic layer on the atomic level such as sputtering and evaporation [28,67,68], galvanisation [69] and electroless deposition [70].

Copper and silver are dominating the metal deposition on fabrics. Normally, electroless copper deposition always needs a conductive pre-activation, for example, using formaldehyde as a reducing agent similar to as shown for yarns with silver nanoparticles as a catalyst [70]. Alternatively, copper can also be deposited by the reduction in copper ions with hydrosulphite. Subsequently, the layer is strengthened by electroplating in a copper sulphate solution [71]. Additionally, conductive polymers, e.g., PANI can be used as a pre-activation layer for a later copper deposition step [72].

## 3. Accelerated Functional Ageing

In order to investigate the ageing behaviour of functionalities and to be able to predict the usage lifetime and safety of e-textiles, accelerated functional ageing tests are often applied. Depending on the usage conditions, different types of accelerated functional ageing are discussed in this section: mechanical ageing, chemical ageing, ageing by washing and laundry and electrical ageing. The review also includes some examples of ageing behaviour of the conductive structure on non-textile substrates, e.g., flexible films, in order to highlight the general damage mechanism under usage conditions.

### 3.1. Mechanical Ageing

Functional ageing because of mechanical strain is one of the most important and most widely investigated ageing categories. Derived from common use, there is stretching, bending and abrasion. The mechanical ageing tests consist of cyclic treatments at a defined intensity. The damage does not occur immediately but is cumulative. Therefore, it is necessary to follow its emergence in order to investigate the causes.

#### 3.1.1. Stretching

**Figure 2:**
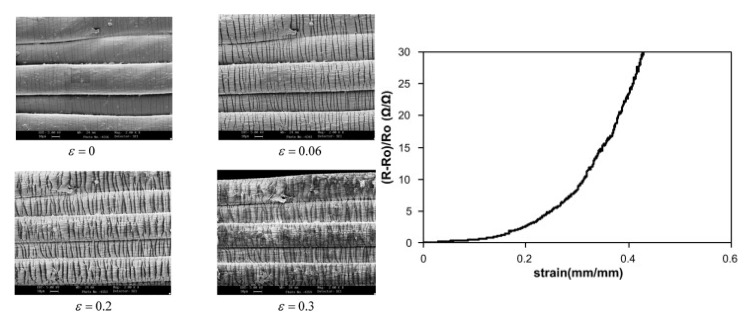
SEM images of crack formation (**left**) and electrical resistance (**right**) during stretching of PPy-coated Lycra fibres; reprinted from [77] with permission from Elsevier.

Stretching is possibly the most common applied mechanical stress to investigate the aging behaviour of e-textiles. However, in order to understand the ageing behaviour of e-textiles, their non-ageing behaviour (c.f. Section 5) needs to be understood as well. Both effects need to be considered separately in order to be able to explain the behaviour at the aged state. The gauge factor describes the relationship of elongation and electrical resistance (c.f. previous section). An increasing electrical resistance during elongation [73] is due to conductive particles or fractions of a cracked conductive layer moving away from each other [74]. The effect can be observed for conductive coated yarns or fibres by microscopy with an accompanied resistance measurement. An example was reported by Li et al. for a polyurethane core with a silver/graphene particle sheath and silicone protection coating [75]. Further studies used SEM imaging for the observation of the changes in conductive coating layers on textiles [76,77]. An example of the crack formation of the conductive layer and resulting increases can be seen in Figure 2 for PPy-coated Lycra fibres [77].

The role of the silver content in gauge behaviour was shown for screen-printed patterns of silver-coated polystyrene particles on a PDMS matrix. The resistance increased with elongation at a low silver content. A rising silver content led to a decreasing gauge factor. Hu et al. explained this observation by the Poisson effect that causes lateral contraction. This means, there is a reduction in the diameter of yarns or fibres while stretching. Hence, the conducting particles are moved closer together [78]. If the formation of new contacts predominates, the electrical resistance decreases during elongation. A descriptive example was published by Sadequi et al. by using “puffy threads” that were composed of polybutylene terephthalate dip-coated with a polydimethylsiloxane/carbon ink. Microscopic imaging during elongation showed decrimping and forming contacts of the fibres followed by a decrease in the electrical resistance [79]. Similar observations were reported for an electroless copper-plated cellulose fabric [9] (c.f. previous section).

The fabric structure plays a role in the non-ageing behaviour as well. Knitted textiles show three stages of stretching, dependent on strain: (1) slippage of the loops that causes variation in the contact points, (2) deformation of the yarn loops and (3) stretching of the yarns [80].

The methods of accelerated stretch ageing are simple and effective and, thus, favoured for simple durability evaluations. Frequently, cyclic stretch tests are aborted after a certain number of cycles, showing no or low damage. Examples for aborted tests are presented for carbon derivative-filled thermoplastic polyurethanes. They underwent 2000 test cycles of 10% or 20% of stretching with no ageing related change of the electrical resistance [81]. A carbon polydimethylsiloxane blend was stretched for 30 cycles at different strain rates without changes in conductivity [82]. Other examples are given for stretchable heat fibres consisting of a polyethylene core, copper nanowires and a silicone rubber protection coating [83]. However, also weft knitted and carbonised modal fabrics showed durability without ageing for 1000 cycles stretched up to 50% [84]. The aborted experiments show durability only for the performed cycle counts. However, as no analytical lifetime investigation is possible, it prevents comparisons with other conductive textile inventions.

A full break investigation requires the occurrence of damage. This is exemplarily presented for silver-sputtered plasma pre-activated PET fibres. Cyclic elongation to 5% led to an increasing mean electrical resistance and a wider distribution of the resistance values, i.e., a higher gauge factor, with rising cycle numbers. After 3500 cycles, first signs of material fatigue occurred. The treatment was stopped at 5000 cycles. A further failure investigation included SEM imaging, showing cracks and waves on the silver surface that were not present in the initial state [85].

Cyclic stretch durability testing is a possibility to compare different manufacturing methods. Koshi et al. investigated the influence of the permeation depth of a stretchable silver ink onto the ageing behaviour. Inks with two different viscosities were screen printed onto plain woven cotton fabrics. The printed conductive lines contained a gap with a resistor mounted with either an electrically conductive adhesive or a soldering paste. The different samples underwent cyclic elongation to 5%. The resistance of the e-textile was measured in situ. Beside the oscillation due to the gauge behaviour, the electrical resistance increased with an increasing cycle number up to a factor of 100. The e-textile with deep ink permeation and soldered contacting showed the best durability. SEM imaging illustrated the cracks and especially their propagation next to the contacts. Young’s modulus of the mounting material is important for this type of connection geometry, to ensure the dissipation of the physical forces at the connection. Additionally, shearing forces were applied on the overhanging resistors. The soldered connections again showed the best durability [86].

The influence of relative textile direction and the dimension of printing patterns on functional aging was also investigated by Koshi et al. They printed a silver containing ink onto plain woven cotton fabrics parallel to the textile yarns and at a 45° angle between the warp and weft yarns. The width of the conductive lines was of either one or three yarns. All samples underwent cyclic elongation to 5% while measuring the electrical resistance with the four-probe method. The samples printed at a 45° angle fatigued faster because of the force distribution at the yarn crossings during stretching. The broader conductive lines survived longer due to the effect of the redundancy of pathways. Despite cracks in the coating, the current still had the possibility to take an alternative way around the damage. With the breakage of thin conductive lines, the electrical current is completely interrupted. SEM imaging after the first cycle and after the 100th cycle of stretching proved the explanations. They also showed an increase in number and width of the cracks for higher cycle numbers. The observations showed a high increase in resistance for the first stretch cycle after manufacturing. This is explained by yarn friction that hinders the displaced yarns from returning to the original state [87].

**Figure 3:**
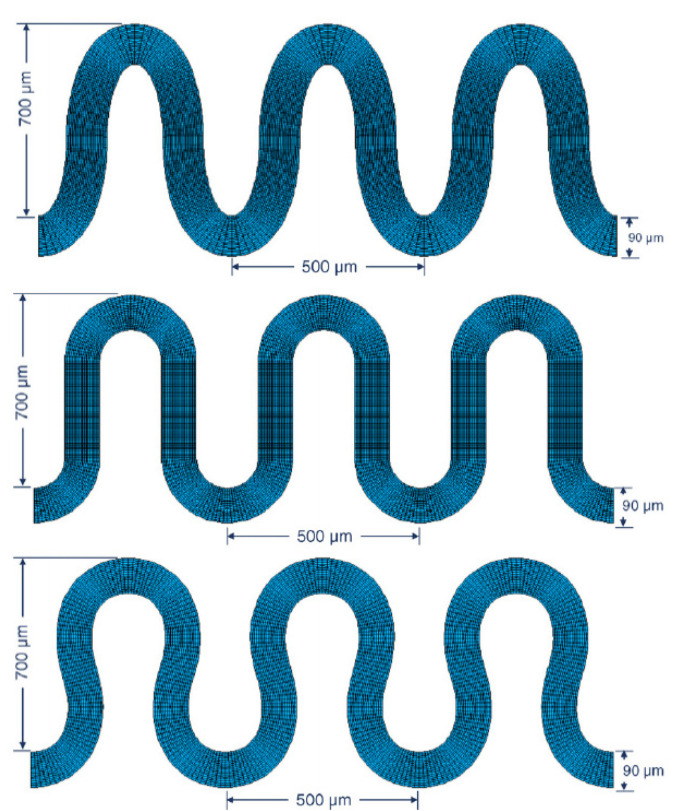
Investigated meandering patterns by Gonzales et al., elliptical, U-shaped and horseshoe-shaped; reprinted from [89] with permission from Elsevier.

Another group investigated the fatigue behaviour of different meandering shapes using a double track approach. Before the realisation of material experiments, the behaviour of patterns was predicted by a finite element simulation. Figure 3 shows three investigated patterns—“elliptical” shaped, “U” shaped and “horseshoe” shaped. The simulation showed the distribution of the mechanical stress during stretching. For the material implementation, nickel and gold layers were encapsulated by PDMS films with the aid of a photoresist in the depicted patterns. The simulation as well as the cyclic load application experiments showed the best durability for the horseshoe-shaped patterns. The latter showed the best distribution of mechanical forces during elongation. A sample version with multi-track patterns consisting of four parallel thin tracks instead of one wide conductive line, showed still a higher durability. Despite the film model substrate, the effect of patterns on the mechanical ageing can be clearly demonstrated by both modelling and experimentally [88,89].

Taking a similar approach on film substrate, Bossuyt et al. applied horseshoe-like patterns on different flexible substrates, e.g., copper foil on PU or PDMS, or silver-containing paste on nonwoven textiles. The maximum tensile load was determined ahead of durability tests. Then, the samples underwent cyclic load experiments with a statistical fatigue evaluation. Different elongation values were applied, i.e., 2.5%, 5% and 7.5% for the copper foil on PU and additionally 4% and 10% for the copper foil on PDMS. The lifetime was defined as 63.2% of the cycle number of total failure. The course of the resistance during cyclic elongation showed a sharp transition from functioning to failure, i.e., a “sudden fatigue”. The numbers of cycles to failure were used for a statistical evaluation on the basis of Weibull functions. The evaluation demonstrates a cumulative damage behaviour for each level of strain (c.f. Section 4). Statistics revealed the assignment of the fatigue function to the Manson–Coffin relation for lower fatigue cycle numbers, i.e., high strain. Here, the deformation of the sample is plastic. Higher fatigue cycle numbers, i.e., lower strain, are assigned to the Basquin relation belonging to the elastic behaviour. A combination of both parts is stated as:

(1) $$\frac{\Delta \varepsilon }{2}=\varepsilon_{f}{\left(2N_{f}\right)}^{c}+\frac{\sigma_{f}}{E}{\left(2N_{f}\right)}^{b}$$

with Δ*ε* as the total strain range in a cycle, *N_f_* as the mean number of cycles to failure (=cyclic lifetime), *c* as the strain exponent, *ε_f_* the as failure strain, *σ_f_* as the failure stress and b as the stress exponent. The exponents and coefficients are material properties. The statistical results showed very good modelling of the fatigue behaviour and the potential for predicting the lifetime of e-textiles under defined conditions. Optical microscope imaging showed cracks of the conductive lines as material damage [90].

For application on a textile substrate, Isaia et al. knitted a blend of 20% Inox steel staple yarn and 80% PES to form a textile specimen, forming a kind of horseshoe-shaped structure. The specimens were elongated to 16% for 250 cycles with an in situ measurement of the electrical resistance by the four probe method. The course of the electrical resistance showed an increasing mean resistance in combination with an increasing gauge factor, both leading to saturation. This change was attributed to the shift of the contact points of the loops (c.f. resistor model). Microscope imaging in combination with a loop length measurement revealed a clear increase in the loop length comparing the initial and final state. As no material damage was found, the observations were identified as preconditioning. The preconditioning showed stability of the electrical resistance values with restarting the experiment, i.e., the preconditioning was permanent [91].

Other possibilities for enhancing the stretch durability of conductive textiles are pre-stretching during manufacturing and micro-patterning. On a PDMS film as a flexible model substrate, Jones et al. successfully reduced the aging affect by stretching the substrate during the manufacturing process to 25% elongation with a subsequent deposition of a chromium and a gold layer. For testing, samples were either stretched once to 100% or in slow cycles to 15%. The electrical resistance values showed a higher stability, i.e., lower value fluctuation, for the pre-stretched samples in comparison with the unstretched samples [92].

Another example of pre-conditioning was investigated by Graz et al. on PDMS film coated with a 50 nm gold layer. The samples underwent cyclic elongation to 20% with an in situ measurement of the electrical resistance. Initially, the resistance increased with elongation and decreased with relaxation. For higher cycle numbers, the peak shape of the resistance changed to a double peak, i.e., with a drop of resistance in its middle for high elongation. The cyclic elongation caused the formation of cracks and, thus, gold-layer islands. These islands were gaining distance while stretching. Lateral contraction led to new contacting and a lower resistance at high elongation. The breakage of the gold layer into micro gold islands stopped at a certain island size. The electrical properties then remained stable for a further elongation treatment. Taking this stability into consideration, the initial distress cycles can be applied as a pre-conditioning method to minimise later ageing during a possible application [93].

The possible recovery of conductivity after cyclic stretching can be performed by heating in certain polymer/conductive material blends. Inoue, Itabashi and Tada found this special effect for silver-containing polyurethane pastes with an 80% Ag content that were printed on textiles. The textiles underwent 10% elongation for 5000 cycles and showed an increase in resistance from 10^1^ to 10^3^ Ω together with an increasing gauge factor. Afterwards, the textiles were annealed at 100 °C for 10 min and the resistance showed a nearly full recovery, in the case of having no cracks in the conductive coating. Furthermore, the recovery process was repeatable. This kind of functional ageing is called reversible or recoverable functional ageing. As a possible mechanism of the observed regeneration, a rearrangement of the polymer structure was assumed [94,95,96,97].

#### 3.1.2. Bending

Cyclic bending plays a similarly prominent role as cyclic stretching. The variety of bending methods is very broad. The bending angle and bending radius are two of the main parameters and low bending radii lead to sharp bending. Bending experiments sometimes also contain additional elongation. Bending, per se, is stretching at a gradient through the textile plane. In the inner part of the bending angle, the stretching factor becomes negative and the textile is compressed. Komolafe et al. used the basic beam bending theory for the calculation of the ideal position in a multilayer system to obtain the stretch factor to 0. The authors printed conductive tracks onto fabrics and encapsulated with polyurethane. The calculated thickness of the encapsulation shifted the conductive track into the calculated neutral axis [98]. Basically, the principle of the beam bending theory is based on the stress distribution over the sample cross-section during bending, i.e., one sample side is loaded with tension while the other side is loaded with compression, with the basic assumption of a homogeneous material. The application of the theory on textile substrate, however, has limitations, for example, because of the different elastic moduli of fabrics in tension and compression. Furthermore, conductive coatings also influence the very different movement behaviours of yarns and fibres in a textile structure (e.g., knitting vs. weaving), leading to changes in the elastic behaviour of the fabric substrates.

Guo et al. investigated accelerated aging by bending for Cu, Ag, Au and Ni electroless-coated PET and PI film substrates. Although the substrate does not represent a typical textile structure, it is a good way to separately investigate the ageing and damaging behaviour of the coated conductive layers. The polymers were activated by plasma and 2-(methacryloyloxy)ethyl-trimethylammoniumchloride (METAC) with the subsequent printing of a (NH_3_)_2_PdCl_2_ catalyst ink. The specimen underwent bending tests with an angle of 180° and different radii. The samples were turned to stretch or compress the metal coating. The performance of 4000 cycles showed no resistance change for most samples [99]. In another work of Heo et al., silver nanowires were drop-coated on a PES-Spandex textile composite with PDMS to create a stretch sensor. No aging affect was observed after 2000 bending cycles with an angle from 0° to 150° [100]. These examples on polymer films demonstrated the use of the accelerated aging test method to prove the reliability of conductive coatings within a certain strain range. As no ageing occurred, the reliability is only valid for the performed low cycle numbers and no further damage or statistical evaluation is possible.

Stempien et al. printed a silver ink with a valve jet print head onto polyacrylnitrile, polyethyleneterephthalat, basalt, cotton, cotton/PET blend, aramid, wool and polypropylene (nonwoven) followed by a curing step at 130 °C. The samples were attached to a moveable and a fixed clamp and bent for ±30° out of plane for 50 k cycles. The edge of the moveable clamp functioned as a sharp bending line. The resistance was measured in situ by contacting the clamps. Rearrangements in the yarn system increased the electrical resistance for low cycle numbers and crack formation increased the electrical resistance and gauge factor for higher cycle numbers. The beginning of a significant increase in the electrical resistance was defined as the fatigue of lifetime. It ranged from 800 cycles for the polyacrylonitrile substrate to 50 cycles for the PET substrate [101].

De Kok et al. performed three related mechanical tests, i.e., stretching, bending and dynamic flex testing with different kinds of samples. For a start, a 1 µm Ag-coated nylon 66 single yarn was stretched cyclically. Increasing the cycle numbers led to an increasing electrical resistance and an increasing gauge factor. The increasing resistance was explained by the ongoing delamination of the silver coating, i.e., a loss of conductive material. For the aged state, this also meant interruptions of the conductive layers on the fibres and, by this, an interruption of the percolation. The increasing gauge factor was explained by the lateral contraction of the yarns while stretching, moving the fibres in the yarns together. The newly formed contacts lowered the resistance. This phenomenon is more effective for the aged state, as gaps in conductive layers of single fibres are bypassed by parts of functioning neighboured fibres. The stretching experiment was repeated with PU-coated filaments of the Ag-coated yarns. The additional coating prevented the formation of new contacts by lateral contraction. The electrical resistance increased with elongation contrary to the decrease for the unprotected samples.

The conductive yarn was applied on PET nonwoven fabrics by stitching embroidery and by TFP. For bending tests, the specimens were attached to springs at both ends. With a speed of 38/min a rod of 10 mm diameter deflected the middle of the sample to 80 mm below original height with a force of 20–25 N. In this process, the textile was elongated by about 0.5%. A total of 5000 cycles were performed with a 10 min interruption after the 500, 1000, 2000 and 4000 cycles. The course of the resistance showed an increasing mean resistance together with an increasing gauge factor. Both led into a saturation level. The 10 min interruptions showed an instable short-term recovery of the resistance. Among the sample manufacturing variety, TFP showed a better ageing performance than stitching embroidery.

Dynamical flex testing is a derivative of bending tests. The bending angle is fixed at 90°. The specimen is pulled over a corner, consisting of an insulated roll of 20 mm diameter. By this, bending effects the whole textile. A pulling force of 12.5 N was applied. One whole cycle comprised rolling back and forth. Within cycling, the experiments were paused with a regeneration time and initiated again. For flex testing, a silver coated nylon yarn was embroidered into different patterns. No correlation between the shape of the patterns and functional ageing was found. The behaviour of the electrical resistance in dynamical flex testing showed a similar trend as the bending ageing. After the experiments, the yarns were extracted from the textile for evaluation. Stitching embroidery exposed the yarns to the roller. This led to more damage on the inside than on the outside. The TFP textile showed less damage for two reasons. The yarn was mechanically less strained during fabrication and the yarn was placed on the outside of the roller during testing [50].

De Vries et al. applied the deflection method described above for the e-textile contacting investigation. For sample preparation, an LED was mounted on a siloxane substrate on a plain woven cotton fabric with a silver-coated copper wire power supply. A second fabric was attached on top with a cut-out for the LED. In a second version, a hard polycarbonate shell was attached on the LED before covering with the second textile. In the third version, a third textile layer was embedded between the hard shell and the power supply. The specimens were deflected with a polymer rod with gaps not to touch the LEDs. The electrical current was measured in situ. The samples fatigued with a sudden drop in the current. The cycle number until fatigue was used in a statistical lifetime evaluation with the help of Weibull distribution for the determination of the unreliability *F(t)*:

(2) $$F\left(t\right)=1-e^{{\left[-\frac{t}{\eta }\right]}^{\beta }}$$

The shape parameter *β* is defined by the type of failure, i.e., early failure, random failure or wear out failure. *η* as the scale parameter is the number of cycles to failure. The location of breakage was analysed by X-ray imaging. The first sample showed the shortest fatigue lifetime with a breakage at the soldered connection next to the LED. The encapsulation stiffened the textile and shifted the point of inflection and breakage from the soldering connection to the edge of the encapsulation. The third version with the textile inlay showed the best fatigue behaviour, as the hard edge of the polycarbonate capsule was softened by the additional textile layer [102].

The previous described procedure was conducted in a variation. ELEKRISOLA litz wires were woven into a polyester fabric with floats for contacting LEDs with a conductive adhesive. The deflection method was applied with a frequency of 40 /min and different magnitudes of deflection. The breakage of the wires led to a sudden drop in the current. The bending radius was estimated as the function of deflection, bending angle and geometry. By this, the connection of the number of cycles to failure with the bending radius was drawn. The Manson–Coffin relation provided a mathematical evaluation for the high cycle fatigue problematics with the possibility of the calculation of the remaining lifetime [103].

With a decrease in the radius and an increase in the bending angle, bending becomes folding [99,104]. Thus, folding can be described as a “severe form of bending” [105]. A simple folding method was conducted by Cai et al. for cysteine-functionalised cotton fabrics with adsorbed Ag nanoparticles and a hydrogel protective coating. Folding was performed by hand, with an additional 100 g weight placed on the fold line. This method was cycled 80 times. The electrical resistance increased until reaching a saturation value. The experiment showed the positive effect of the protective hydrogel coating onto the electrical lifetime [106].

The hand-folding method was also applied in a variation by Zahid et al. on a plain woven cotton fabric, spray-coated with PEDOT:PSS and graphene nanoplatelets. The specimen was folded 180° and a 0.5 kg metal weight was placed for 1 min. After bending to 0° and relaxing for 1 min the electrical resistance was measured. The folding was repeated for 20 cycles showing an increase in the electrical resistance. Small cracks in the folding line were observed with an optical microscope [105].

An example for the mechanisation of folding is shown by Lim et al. An impacting machine with a crank applied a pressure of 15 kgf/cm² onto a folded fabric at its minimum height excursion. A textile capacity pressure sensor was prepared from a PEDOT-coated nylon/polyester microfibre fabric. The sensor was folded for 1000 cycles and tested with a reference weight in between [107].

Lee et al. used a folding endurance tester. The specimen was attached to the machine at three points. The first attachment was at the static part of the machine, the second in the middle of a rotating disk and the third at the edge of the rotating disk. The disk rotated back and forth to cover nearly 360°. A conductive silver ink was printed on PU-coated cotton fabrics and encapsulated. The treatment with the MIT folding endurance tester showed a clear ageing advantage for the encapsulated samples [108].

Stretching and bending showed in some parts similarities. As mentioned, bending contains some kind of stretching, mostly with a gradient. Hence, some conductive textiles show the similar resulting damage formation. The elongation in combination with different Young’s modulus leads to at least breakages of the conductive layers.

#### 3.1.3. Abrasion

Abrasion is the third important part of the mechanical stress testing on e-textiles. The test methods are simple in execution and mainly used to prove the durability of inventions, often accompanied with other accelerated ageing methods. Examples for simplified abrasion experiments on conductive textiles are given in [109,110,111,112,113].

Stempien et al. investigated the effect of the abrasion direction onto printed conductive lines in dimensions of a 14 cm length and 3 mm width on different substrates. The rubbing finger of an AATCC crock meter for testing colour fastness was prepared with a cotton cloth as the abradant. The textile was abraded for 2000 cycles in the printing direction and crosswise. The abradant was replaced every 200 rubs. The electrical resistance was measured in situ. The lengthwise abrasion caused a faster fatigue due to a higher loss of material. Additionally, the integration of the conductive ink into the substrate material played a role for the rubbing durability of the conductive lines [101].

**Figure 4:**
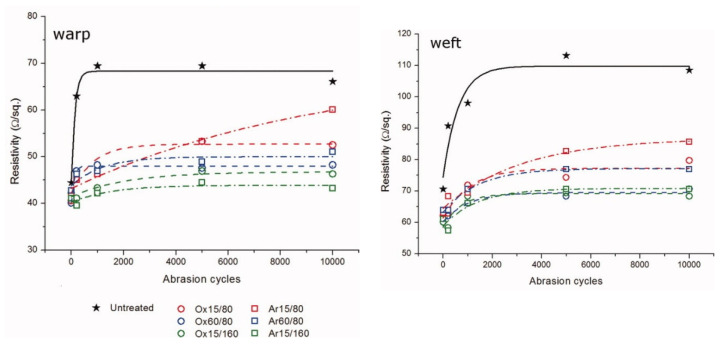
Behaviour of the electrical resistance in warp direction (**left**) and weft direction (**right**) during abrasion of PPy-coated polyester (PET) fabrics [114]; reprinted with permission from Wiley.

The influence of plasma pre-treatment on the abrasion stability of the coated textile was investigated by Montarsolo et al. They utilised radio frequency-induced low-pressure plasma to activate a woven PET fabric at two different power stages. Additionally, reaction times and the process gas (oxygen and argon) were varied. The pre-activated fabrics were coated with polypyrrole. Martindale abrasion testing according to EN ISO 12947 was performed with samples of a 15 cm diameter and a pressure of 9 kPa for 10,000 cycles. Warp and weft yarns were examined separately. Despite their different absolute electrical resistance values, the same ageing behaviour was observed for both directions. The resistance graphs showed a fast increase for low cycle numbers, leading into saturation. The plasma-treated samples outperformed the untreated ones. Samples activated under harsher conditions showed the best abrasion durability for both fabric directions (Figure 4) [114].

Further examples for the application of Martindale abrasion testing on e-textiles are described in [115,116,117].

An increase in the abrasion harshness due to the textile–sandpaper friction was performed by Bogan et al. The researchers tested a woven nylon fabric with a different content of twisted enamelled copper yarns in the warp direction. The Martindale method with a load of 9 kPa and standard worsted wool abradant did not cause damage over 50 k cycles. A Wyzenbeek apparatus was used for the heavier-duty stress. An 800-grit sandpaper functioned as the abradant and was applied onto the tensed sample. The electrical resistance was measured in situ. The course of the resistance showed sudden fatigue, i.e., an interruption of the conductivity. A lower number of conductive yarns per track and a lower content of non-conductive yarn in the twisted yarns showed an earlier lifetime fatigue. The failure of the conductive lines appeared together with the failure of the textile substrate [118].

### 3.2. Chemical Ageing

Chemical ageing is considered when exposing e-textiles to chemical environments, e.g., water and aqueous solutions of detergents or bleaching agents during the wash/laundry process. However, chemical ageing also comprises the influence of oxygen from air or the attack of acids, bases or solvents. The influence of the wash/laundry process on the functional aging will be discussed later in Section 3.3 as the process comprises both mechanical and chemical ageing.

However, the storage on the air atmosphere already affects oxygen-sensitive materials such as conductive polymers. For storage stability testing, Maity et al. coated a nonwoven textile substrate with polypyrrole. Storage at 25 °C and 65% RH for 18 weeks showed an increase in electrical resistance of approximately 10%. The ageing was attributed to de-doping of the conductive polymer because of oxidation [119]. Moss et al. [120] and Tabaciarova et al. [121] investigated the oxidation reaction of polypyrrole by means of XPS and IR spectroscopy. Additional carbonyl, aldehyde and hydroxyl functional groups were found on the polymer. The low activation energy of free radical mechanisms led to the reaction with oxygen from air. A high oxidation sensitivity during storage on air was reported for PANI, showing an increase from about 500 Ω/5 cm to a saturation level at 3000 Ω/5 cm [22]. Conductivity loss because of oxidation is also reported for PEDOT:PSS [122].

Relative humidity and temperature define the storage climate. Varesano et al. varied both to find out about their impact onto the defunctionalisation of polypyrrole-coated PET fibres. Whereas the change of the relative humidity had no or only a low impact, an increased storage temperature up to 80 °C yielded in a fast gain of electrical resistance. In counter, storage at −28 °C did not significantly slow down the ageing in comparison to ambient conditions [123]. A protective coating was able to prevent oxidation by air, e.g., shown for liquid latex [124,125].

Storage ageing on air also impacts copper particles deposited on functionalised yarns. Liu et al. showed a decrease in electrical conductance of 90% within 7 days attributed to the oxidation of the metal [126]. They also showed a limitation in the increase in the electrical resistance onto the first 48 h after manufacturing, with a remaining constant saturation value over two weeks [127]. Hwang et al. printed ink containing copper nanoparticles or copper nanoparticles with additionally multi-walled carbon nanotubes onto a polyimide foil using the doctor blade method. The ink was flashlight sintered by xenon light. The samples were stored on air at 25 °C and 30% RH over a period of 30 days. The electrical resistance increased over time due to the oxidation of copper to copper(I)-oxide that was found by an XRD analysis. The ageing was accelerated by increasing the temperature to 85 °C and relative humidity to 85% over 14 days, showing a faster gain of electrical resistance [128].

In order to investigate the influence of acid, base and salt on ageing, Ali et al. immersed electrochemical copper-deposited fabrics in 0.3 M hydrogen chloride, 0.3 M sodium hydroxide or 50 g/L sodium chloride for 24 h. All solutions increased the electrical resistance with hydrochloric acid having the highest impact with 50% of increase. The impact of the sodium hydroxide solution onto the electrical resistance was lower, followed by the sodium chloride solution [71].

**Figure 5:**
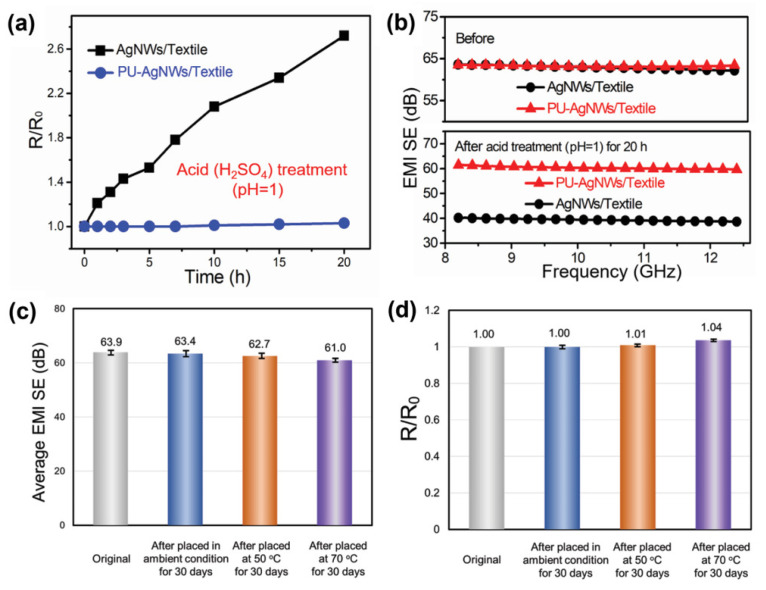
Effect of chemical ageing on the electrical resistance of silver nanowire/polyurethane-coated fabric: (**a**) relative resistance (R/R0), (**b**) electromagnetic interference (EMI) shielding, (**c**) EMI shielding before and after placed at different conditions, (**d**) R/R0 before and after placed at different conditions [129]; reprinted with permission from Wiley.

To test the protection efficiency against chemical ageing Jia et al. coated a tricot-knitted textile with silver nano-wires and a polyurethane protective layer. Samples of the conductive textiles were immersed in sulphuric acid at pH = 1 at 25 °C for 20 h. Unprotected samples nearly tripled their resistance and decreased the EMI shielding about one third in the range of 8 to 12.5 GHz, while the PU-protected samples showed no changes (Figure 5) [129]. An alternative for polyurethane protection is polydimethylsiloxane [130].

Erdogan et al. tested the impact of acids and bases onto polythiophene and polyaniline-coated PET yarns. The samples were immersed into hydrogen chloride or ammonia solutions of pH 5 to 11 at RT. After 1 h, they were rinsed and dried at 50 °C. The electrical resistance of the polyaniline coating strongly increased, especially for higher pH values. A mixture of polyaniline and polythiophene showed the lowest electrical resistance for pH 9 and below. The values ranged in the area of 10^6^ Ω, as the conductive polymers were not doped. The resistance changes were explained by protonation phenomena in the pi-system [131].

Considering the exposure of wearables to body sweat, Priniotakis et al. manufactured woven, nonwoven and knitted ECG electrodes from stainless steel fibres. The electrodes were attached to a PTFE membrane with pores of 5 µm, simulating the skin. A mounted PVC tube contained artificial sweat. The electrochemical impedance between the electrodes was recorded. For short-term experiments, differently concentrated sodium chloride solutions showed a reduction in the electrical resistance. The artificial sweat for long-term experiments contained 50 g/L NaCl, 1 g/L urea and 0.5 g/L of other salts. The pH value of 5.8 was adjusted by NaOH and HCl. In the first 100 h the resistance increased very fast from 250 Ω to about 400 Ω, leading into a saturation for the next 400 h [132].

Another important aspect of chemical ageing is the influence of organic solvents, for example on conductive polymer coatings. Gosh et al. immersed a PEDOT:PSS-coated cotton fabric for a not closer stated time into boiling water, ethanol, toluene and tetrahydrofuran. Afterwards, the EMI shielding was tested in a range of 8 to 12.5 GHz. Boiling water lowered the shielding values slightly. The other solvents did not show a change compared to the initial state [133].

Han et al. immersed polypyrrole-coated aramid nano-fibrils into acetone, tetrahydrofuran, ethanol, dimethylformamide, glycol and glycerol. The electrical resistance was measured after 24 h and 175 h. The solvents showed a low impact. The highest effects occurred for acetone after 24 h and for ethanol after 175 h which increased the electrical resistance with a factor of about 2.5 of the initial state [134].

Chemical ageing has by far not been investigated as exhaustively as mechanical ageing, especially considering the long-term effects of the impact of water or sweat being important for wearables. However, chemical ageing plays a big role in wash ageing too, as there is an impact of water, detergents and additives.

### 3.3. Ageing by Washing and Laundry

Washability of conductive textiles is a striking challenge. It contains several single ageing compartments. Water, detergents and additives cause chemical ageing, and motion causes bending and abrasion. Several approaches and test methods have been developed to simulate the durability of e-textiles during the washing process.

In simple methods, conductive textiles or sensor specimens are immersed and stirred in water or detergent solutions. The results are difficult to transfer to machine washing [135]. As an example, gold-coated knitted polyester fabrics were stirred for 1 h in laundry detergent and dried at 60 °C overnight. The testing was stopped after a few cycles with no ageing effect [136]. Another example for beaker wash testing is shown for a PEDOT:PSS coating. The testing performed three cycles [137]. Additionally, the handwashing of copper-deposited samples [138] or the usage of an oscillating dyeing machine for a reduced graphene oxide-coated fabric [139] were described. All these methods, as described, do not achieve the harshness of a domestic machine wash.

In a different approach, Schwarz et al. coated activated para-aramid yarns by copper deposition and applied the wash durability testing ISO 6330:2000 standard for an ageing study. A domestic washing machine was loaded with the samples and 2 kg of cotton fabrics as dummies. The load underwent 25 washing cycles at 40 °C for 40 min each. The electrical resistance was measured by the four-probe method after 5, 10 and 25 cycles. The values increased, but the fabrics were still considered as functioning. SEM imaging showed damaging and the delamination of the conductive layers [33,140].

**Figure 6:**
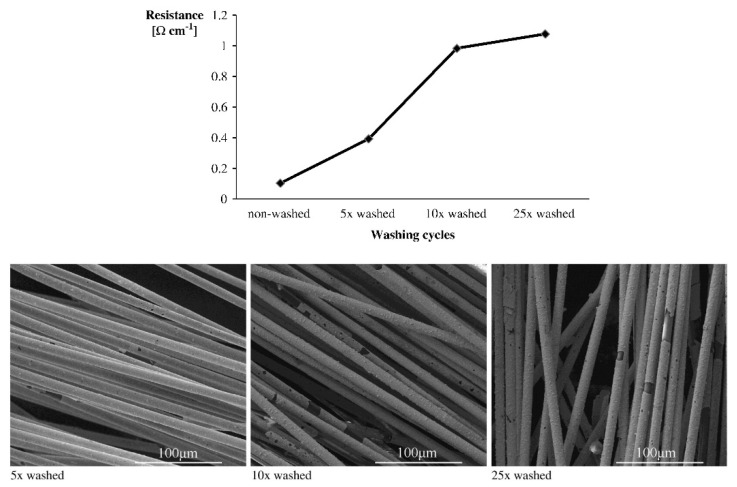
Increase in electrical resistance (**top**) and damage of coated conductive layer (**bottom**) after several washing cycles of gold-coated aramid yarns [140] reprinted with permission from Elsevier.

An example on damaging of conductive coatings and changes in resistance is given by Schwarz et al. [140] in Figure 6 for gold-coated aramid yarns after several washing steps.

A further example for the application of the ISO 6330 standard is given for ECG electrodes composed of different PEDOT:PSS-coated fabrics. The electrical resistance was measured after each washing cycle. Despite an increase in the resistance, samples showed durability for 50 washing cycles [141]. In a similar simplified wash durability test, samples underwent cyclic washing and damages were visualised by imaging afterwards [109,142]. Additionally, standardisations from the American Association of Textile Chemists and Colorists (AATCC) were used for wash durability testing [143,144].

Considering washing/laundry as a combination of chemical and mechanical ageing, both processes contribute to the ageing of e-textiles. The effect of washing and its simulations onto silver-coated yarns was extensively investigated by the working group of Vladan Koncar. Zaman et al. used commercial and self-made textile ECG electrodes for the investigation in both ageing compartments. The commercial electrodes consisted of knitted polyester fabrics with a copper, copper/nickel or silver-coating. The self-made electrodes consisted of embroidered silver-coated yarns [145]. The resistance was determined by a four-probe measurement. Wash durability tests were performed according to the ISO 6330 standard domestic washing. The programmes “silk” and “express” were used with the main difference in the rotation speed of 15 rpm and 38.5 rpm. The spinning intensity was 400 rpm and the runtime 35 min at 40 °C. The amount of detergent was 20 g for a concentration of 1.25 g/L for a 2 kg load. In total, 50 wash cycles were carried out. The experiments were repeated four to six times.

The simulation of the mechanical part of wash ageing contained abrasion and pilling tests. The abrasion experiments were performed on a Martindale apparatus with a standard wool abradant, 9 kPa pressure and 10k back and forth rubs. For the pilling tests, the electrodes were embroidered to cotton fabrics and attached to tubes. The tubes were treated at ambient conditions at 60 rpm for 10k cycles in an orbiter pilling and snagging tester. The simulation of the chemical ageing consisted of a motionless immersion into solutions with a detergent concentration of 1–1.25 g/L. The time periods were varied from 30 min to 72 h at a temperature of 40 °C.

The silver-coated electrodes showed no ageing for 50 wash cycles for the “silk-wash”. The other samples showed a slight increase in the electrical resistance. In the “express-wash”, the silver electrodes were stable as well. The copper and nickel coated samples fatigued within the first 10 cycles. Frequency scans from 0 to 100 Hz proofed the functionality of the surviving samples. SEM imaging documented the damage as the delamination of the metal coatings. In comparison, the silver-coated self-made electrodes also showed wash ageing stability.

The Martindale abrasion testing over 10k cycles showed no gain of resistance for all commercial samples. The friction forces were too weak to delaminate the metals from the polymer. The self-made electrodes showed a slight increase in resistance. In the pilling tests, they showed no ageing as well. The immersing tests showed no impact on the electrical resistance of the silver-coated electrode. The copper and nickel-plated fabrics showed a small increase in the electrical resistance. It was concluded that the copper and nickel layer were oxidised during immersion and, thus, their adhesion to the polymer surface decreased [146].

A similar approach for the chemical part of wash ageing according to ISO 6330 was performed by Ismar et al. for commercially available silver-coated polyamide yarns. The proceeding delamination of the conductive coating was demonstrated by increasing the IR absorption of the polymer substrates [147].

Zaman et al. also performed a further approach for the simulation of the mechanical part of wash ageing. It was performed similar as in the previously described simulation with Martindale abrasion and pilling tests. Two and three-ply yarns were stitch embroidered on plain woven cotton fabrics with different stitch lengths in straight or zigzag lines and as single or three-line conductive path. The yarns were contacted with snap buttons in combination with silver paste. The machine wash treatment showed the best durability for the three-line embroidered sample. Among the ply yarns, the silver-coated yarn plied with two non-conductive yarns showed the slowest increase in resistance. Harsher conditions of “express-washing” were reflected in a faster gain of electrical resistance. The abrasion showed a resistance gain for all samples. The authors established correlations between the course of the resistance of machine-washing and abrasion testing. It was estimated that 1000 abrasion cycles could substitute eight or nine “silk-washes” and five or six “express-washes”. Similar results were obtained for the pilling box test. About 1000 cycles were meant to substitute four “silk-washes” or three “express-washes”. Non-fitting correlations, i.e., for a three-ply conductive yarn, were not taken into account for modelling. Microscopic imaging revealed a delamination of the silver coating as material damage [148].

Atakan et al. recombined the simulation of the mechanical ageing and chemical ageing in wash durability testing. Silver-coated polyamide yarns were subjected to Martindale abrasion in the dry state, soaked with water or soaked with an aqueous detergent solution. The addition of water to abrasion testing accelerated the delamination of the silver layer. The ISO 6630 conform addition of detergents increased the ageing even more, especially for commercially available detergents. The commercially available detergents contained particles that increased friction. The authors concluded that soaked abrasion is a suitable simulation for predicting the machine-washing lifetime of e-textiles [149].

Ankhili et al. tested the wash durability of protections and embroidery techniques against EN ISO 6330 conform domestic machine-washing. Silver-coated polyamide yarns were embroidered in different patterns. Beside convoluted zigzag lines that were crossing each other, two different types of protection were tested. The embroidered conductive lines were either coated with thermoplastic polyurethane (TPU) in a hot-press or covered with protective embroidery with a non-conductive polyester yarn. After 50 cycles of washing at 40 °C followed by 24 h of drying on air, the samples were investigated for change in electrical resistance, SEM-imaging and signal to noise ratio (SNR) in the frequency band 0 to 100 Hz. The unprotected zigzag-embroidered samples showed early fatigue. SEM imaging revealed the delamination of the silver layers as the cause of fatigue for the unprotected samples. The TPU-protected samples and the embroidery-protected samples survived the performed washing cycles with a slight increase in the electrical resistance. The embroidery-protected samples showed the best wash durability. This was led back to a better drying performance after washing. The penetration of water into the TPU-coated samples caused chemical ageing with a prevention of a fast evaporation due to the TPU coating. The electrodes surviving the machine wash testing showed no quality loss for the ECG signals measurement [145].

For bright clothes, bleaching agents with oxidising properties can be applied. Gaubert et al. knitted silver-coated nylon yarns together with cotton yarns into a double jersey fabric. The samples were washed with a bleaching agent containing detergent and with a bleaching agent-free detergent. A total of 30 washing cycles was performed in a domestic washing machine at 30 °C in the “delicate” programme. The samples were examined by electrical resistance, SEM imaging and colour lightness with a spectrophotometer. The lightness of the bleaching agent additive-washed samples decreased with increasing the cycle number. This was due to the formation of dark-appearing silver oxides. The samples washed with detergents only showed a much smaller decrease in lightness. The electrical resistance of the bleaching agent-washed samples was several magnitudes higher than for the reference. SEM imaging and EDS measurements showed the delamination of the silver layer. It was assumed that the formation of silver oxide decreased the adhesion forces on the polymer surface [150]. Similar results were obtained in a study using sodium percarbonate solutions in machine washing with and without detergent. The combination of oxidative chemical ageing and wash ageing led to faster fatigue, rather than each alone [151].

In order to increase the mechanical intensity, a steel-ball-aided wash ageing was investigated by Karim et al. with reduced graphene oxide-coated woven cotton fabrics. Machine wash ageing was simulated by the standard BS EN ISO 105 C06 A1S. The conductive textile was immersed into an aqueous solution of detergent with a concentration of 4 g/L. In total, 10 steel balls were added. The vessel was turned at a temperature of 40 °C for 30 min. The experiment was performed for 10 times and after each cycle, the sheet resistance was measured. It showed an increase with a high slope at the beginning of the treatment [152].

Apart from conductivity, Toivonen et al. investigated the effect of washing onto the performance of textile antennas. The antennas were manufactured by the embroidery of silver-plated yarns onto cotton fabrics. The water compatibility of the antenna was tested by soaking and drying afterwards. Every four minutes during drying, the read range was measured from 700 to 1200 MHz. The read range level was reduced in the wet state due to the high dielectric constant of water. Additionally, the maxima were dulled and shifted to lower frequencies. During drying, the performance of the antenna recovered to its original state. Three different machine washing programmes were performed: 40 °C, low spinning and a low amount of detergent, 60 °C, middle spinning and a middle amount of detergent and 95 °C with high spinning and a high amount of detergent. Both latter wash programmes led to a fast reduction in the transmitted power for different frequencies. The authors concluded that a protective coating was needed for the maintenance of the antenna performance over several washings [153].

A detailed comparison of wash durability testing methods was performed by Rotzler et al. Here, the different standards from, e.g., ISO, DIN and AATCC were discussed with respect to their suitability to e-textiles. The comparison also included different interpretations of the norms and, as a conclusion, the authors clearly addressed the need of test standards for e-textiles [154].

### 3.4. Electrical Ageing

Electrical ageing takes place due to the electrical operation of a device. It only plays a minor role in the endurance testing of e-textiles. Nagaraju et al. coated a commercially available copper-plated PET fabric with nickel–cobalt hydroxide nanosheets. In order to test the electrical current changes, galvanic or galvanostatic charging and discharging was applied. The sheets were cycled with a current density of 10 A/g in a voltage range of 0 to 0.5 V in a 1 M KOH solution. In total, 2000 cycles were performed and the specific capacity was investigated. It decreased from 1400 F/g to 1200 F/g, which was considered as a good stability [155].

**Figure 7:**
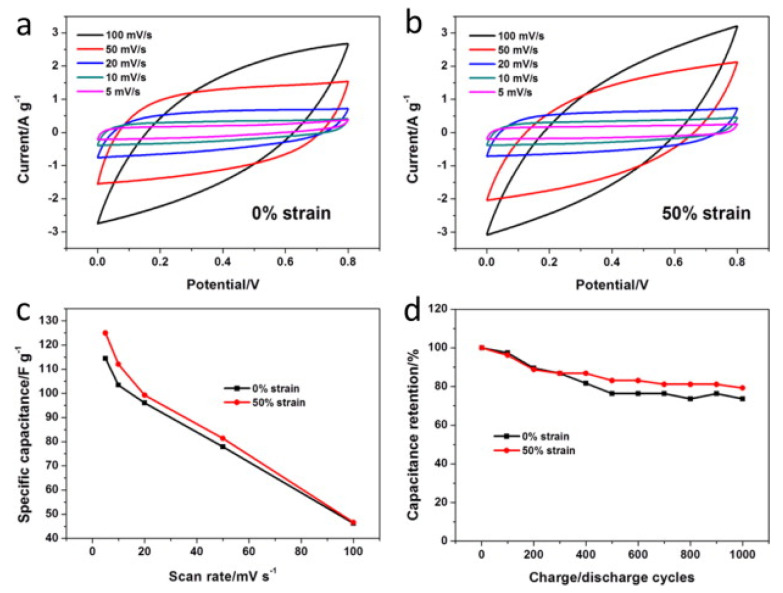
Depiction of voltammetric (CV) curves of graphene oxide/PPy-coated Lycra fabric for supercapacitor application: (**a**) 0% strain (**b**) 50% strain, (**c**) specific capacitances of the at 0% strain and (**d**) capacitance retention of the supercapacitor at 0% and 50% strain. [156]; reprinted with permission from Elsevier.

A further example for an electrical testing method is given by Zhao et al. for super capacitators. The capacitators were manufactured form-reduced graphene oxide or polypyrrole blended with reduced graphene oxide-coated nylon fabrics, attached to nickel foam and separated by a filter paper. The samples were immersed into a 1 M Li_2_SO_4_ solution and cyclovoltammetry was performed in a range from 0 to 0.8 V. The capacitators containing only the reduced graphene oxide showed a stable performance over 2000 voltammetric cycles at about 90% of relative capacitance retention at 50% elongation and about 80% capacitance retention at 0% elongation. The coating of the reduced graphene oxide/polypyrrole blend showed a continuous decrease at both elongation states in the course of 1000 voltammetric cycles to 80% of its initial capacitance (Figure 7) [156].

Another example is spray-coated fabrics with activated carbon ink subjected to voltammetric cycling from 0.8 to −0.8 V over 15,000 cycles. The relative capacitance showed an increase of about 5% for the first 4000 cycles and a slight decrease for the last 2500 cycles [62].

Hana et al. manufactured a copper oxide resistive switch. It consisted of a copper wire woven with a copper oxide-coated copper wire and a platinum spacer between the wire crossings. The switch persisted in a high resistance state until a certain voltage was applied. It then turned into a low resistance state and the current passed. It was used for data storage at a high voltage and data read out at a lower voltage. The presented textile was stable for 1000 readings and 30 switches but the experiments were not continued until ageing or failure [157].

The electrical ageing of conductive textiles was not investigated in depth and only plays a minor role in the literature on e-textiles. This is possibly due to small ageing effects compared to the strong forces of mechanical strains or reactive chemicals. It has not been clarified if electrical ageing is a real cause or just an indicator for other effects such as oxidation.

### 3.5. Challenges in Accelerated Functional Ageing

Overall, activities on mechanical ageing took a big share of research in the accelerated functional ageing of e-textiles. This included major typical loading types in textile application: stretching, bending and abrasion. There is a big difference in mechanical aging behaviour, depending on which technology is applied to the integration of conductive structures into textiles. While embroidery, e.g., TFP with a defined pattern or knitting technique may give a very good stretching behaviour of fabrics, these techniques show limitation in miniaturisation, i.e., generating conductive structures at a fibre level. Metal deposition, on the contrary, may provide a very thin, flexible conductive structure on fibre and textile surfaces. It would, however, need good protection coatings against abrasion and pre-conditioning for a better stretching behaviour. This makes the efforts in modelling more difficult, also when a taking substrate structure (knitted, woven) into consideration.

The biggest challenge in chemical ageing and ageing against washing and laundry is the combination of damages of both conductive structures and textile substrates. Furthermore, washing and laundry also consist of mechanical stresses, so losses in functionality during laundry for example need to be considered as cumulative results of different ageing processes.

Electrical ageing is the least investigated subject in the literature compared to other ageing processes. However, when considering possible electrochemical corrosion during (electrical) operation, this type of ageing deserves higher attention and needs to be investigated with a much higher focus in the future.

At practical application conditions of e-textiles, it is very often, that functional ageing is a combination of all mentioned ageing processes. It remains a big challenge to separately investigate each mechanism independent from each other. Thus, a helpful approach would be the cumulative damage modelling to obtain a combined view on the functional ageing behaviour of e-textiles.

## 4. Modelling of Functional Ageing

To provide a better understanding and forecasting the functional aging process, a mathematical modelling approach is applied. As conductivity is one of the dominant features of e-textiles, electrical resistance or conductance are often used as measures to build models to describe the functionalities (Section 4.1). In another approach, fatigue damage modelling is applied, considering ageing as a cumulative measure over the usage lifetime (Section 4.2).

### 4.1. Resistance Modelling of Conductive Textiles

Theoretical modelling of the electrical resistance in conductive textiles is difficult because of the textile structure and the frequently occurring gauge behaviour. However, there are some efforts in modelling of conductive textiles, e.g., the resistance of e-textiles is modelled as a resistor network (c.f. Section 2). Further examples for modelling of the non-ageing behaviour can be found in the following literature [48,49,78,80,158,159,160,161].

The formation of conductivity in heterogeneous material systems is explained by the percolation theory. The resistance is dependent from the volume fraction of the conductive material in relation to the non-conductive material. Therefore, recording and modelling of a percolation curve is a useful tool in manufacturing conductive fibres, yarns and textiles. This helps to find a fitting content of conductive material within the insulating substrate material and to compare manufacturing methods [162]. An important parameter is the critical volume fraction, which is also called percolation threshold. At this content of conductive material, a conductive path is formed and the resistance drops by some orders of magnitude within a small increase in the conductive material content. The percolation theory in combination with Poisson’s law was used by Hasan et al. to model the course of the electrical resistance during the elongation of a silver particle-coated polyether ether ketone fibre. The change in volume during stretching caused a change in the volume fraction of the silver particles, followed by a change of the electrical resistance [163]. Often, the percolation theory is also drawn for the explanation of evolving damage. A loss of material lowers the surficial concentration of conductive material towards the percolation threshold. This additionally clarifies that not only the concentration, but also distribution is important for percolation.

Empirical electrical resistance modelling of conductive textiles requires the comparison of the resistance at an application condition to the resistance at defined conditions. In order to investigate the influence of a single condition onto the resistance, only this single parameter needs to be changed. An example is shown by Xue et al. for PPy-coated polyamide 6 and lycra fibres. Herein, temperature, moisture, elongation and number of stretch cycles were changed separately, all influencing the electrical resistance. Consequently, ageing caused by both, the strain rate and cycle numbers, was included into the empirical mathematical modelling of the ongoing resistance of the tested yarn [164].

### 4.2. Cumulative Fatigue Damage Modelling

In Section 3.1.1 on stretch ageing, the cumulative character of mechanical damage and a possible lifetime prediction of certain e-textiles were already brought up. On a more general level, cumulative fatigue damage modelling has been established for some material failure problems. The cumulative fatigue damage model describes the changes of materials performances from the initial state to complete failure. Herein, damage is the sum of finite small partial damages that are due to a repetitive cyclic stress treatment. The prediction of the remaining lifetime of an object is a goal of the cumulative fatigue damage theory. The simplest linear cumulative fatigue damage model for a material system includes two different levels of stress intensity. In an earlier stage, such a model was developed by Palmgren [165] and Miner [166]. It is described by the mathematical expression (3).

(3) $$D=F\left(n,r,f,\;T,M,\ldots \right)=\overset{m}{\underset{i=1}{\sum }}\Delta D_{i}=\overset{m}{\underset{i=1}{\sum }}\frac{n_{i}}{N_{i}}=1$$

when the cycle number *n_i_* reaches the maximum number of cycles to failure *N_i_*, the damage parameter D becomes one and the object is broken. This means *D* = 0 for *n_i_* = 0 and *D* = 1 for *n_i_* = *N_i_*. The damage function F is dependent from the cycle number n, the intensity r, the frequency f and other factors such as temperature T or moisture M. Under lab and modelling conditions, frequency and ambient influences are kept constant. Thus, the damage is only dependent from *n_i_*. The ratio *n_i_/N_i_* is also called the fractional lifetime. The damages of different applied stress levels are summed up to obtain the full damage D. The chronological sequence of the different stress levels does not play a role [167]. Fatigue damage modelling does not have to be linear and occurs in a wide variety [166]. For example, the Marco–Starkey ansatz in Formula (4) shows a combination of two stress levels in a test system. The empirical exponent α is introduced to describe the course of the damage accumulation [168].

(4) $$D=\overset{m}{\underset{i=1}{\sum }}d_{i}={\left(\frac{n_{1}}{N_{1}}\right)}^{\alpha }+{\left(\frac{n_{2}}{2}\right)}^{\alpha }=1$$

Cumulative fatigue damage models are often used in lifetime measurements and calculations for composite materials, e.g., reinforced textiles [169].

## 5. Current Practices, Challenges and Development Trends

Despite a high number of publications on the functional aging of e-textiles, there are still many challenges, e.g., in harmonised test methods, the classification of ageing behaviours. In the following sections, current practises, challenges and recent development trends are discussed.

### 5.1. Variation in Test Methods

The functional ageing of e-textiles poses several questions that are related to the choice of material, manufacturing process and testing methods. There are questions on the damage effects and their recording or closer investigation methods but also on the statistical evaluation.

Basically, there are two main reasons for the execution of accelerated functional ageing experiments. The first one is the inducement of material damage with a subsequent investigation of its basic mechanisms for the revelation of its causes. The results can be used for statistical modelling and/or for the further enhancement of the e-textile properties. This has especially been performed for stretch-, bending- and wash ageing. It also includes the development of new test setups, the reconstruction of available systems or the application of standards.

The second goal of accelerated ageing experiments is proofing the durability of new inventions and developments, which covers the majority of the reported literature. Here, the ageing experiments only play a minor role. However, it also provides the possibility for a deeper investigation on a material and statistical scale, leading to an enhancements of the product.

Nonetheless, many functional ageing investigations are strongly minimised and simplified. We distinguish three main problems for these minimised supporting ageing experiments. One is the selection of the ageing category, that is sometimes focused on only one method, despite the availability of other possible tests. Another crucial point is the premature stop of accelerated ageing experiments at low treatment cycle numbers before any ageing occurs. The number of cycles sometimes seems to be chosen randomly. This kind of aborted testing only shows the durability for the performed cycles. However, a lifetime investigation would be better suitable for a fair comparison and for the estimation of lifetime under application conditions. The lack of ageing in some cases may also be derived from the low strain level intensity. Soft ageing methods do not generate material damage and by choosing the right conditions, e-textiles will survive any cycle numbers. This hinders simple comparability with other e-textiles.

As the research subject of accelerated aging is rather new and there are no standardised test methods, a fair judgment of the performance of the functionality as function over time is rather difficult.

We suggest considering the manufacturing process of a conductive textile for deciding on the test methods, apart from the textile substrate, conductive material and protection method. Additionally, the contacts to electrical devices and the intended application of e-textile play a role by defining the suitable test regime.

A good possibility to cope with several problems is the application of standards for classical textiles onto e-textiles. The EN ISO 12947 standardised Martindale abrasion testing works for testing all kind of coatings but also yarn-integrated textiles. Despite several attempts of the simulation of wash ageing, we consider wash testing according to ISO 6330 standard testing with domestic washing machines as the best solution to demonstrate washing durability.

### 5.2. Ageing Classification and Non-Ageing Behaviour

The ageing classes are mainly derived from the experimental testing setup. An example is mechanical ageing with stretching, bending or abrasion as the subcategory. The material damage mechanism does not play a role for the classification, e.g., “delamination-ageing” or “layer-breakage-ageing”. The same is valid for wash ageing. Although comprising mechanical and chemical ageing, it exists as its own category. This is due to its experimental setup that contains only special segments of mechanical and chemical ageing treatments. The separation in mechanical and chemical aging is, however, reasonable and desired for a deeper investigation of the basic mechanisms. For simple durability testing the term wash ageing is more suitable, as, e.g., the number of machine wash cycles to failure is a magnitude for durability.

Chemical ageing is rather complex as it is due to the exposure to reactive substances, e.g., oxygen, acids or bases, but also leads to a change in the chemical composition of the conductive layer in e-textiles. The electrical ageing does not play a big role for e-textiles. It comprises damages due to the electrical operation that seems to play a small role in their extent compared to the other ageing mechanisms.

While the functional ageing was mainly due to material damaging, we summarised all non-permanent kinds of behaviour in the expression “non-ageing behaviour”. Non-ageing behaviour is a direct response to an external influence that lasts for the time of impact with a subsequent full recovery. In contrast to the non-ageing behaviour, the reversible or recoverable ageing is permanent after an external influence but recovery is possible with the application of a further external effort.

### 5.3. Types of Failure

Durability testing of e-textiles is very often performed until failure occurs. There are several possibilities being considered for the indication of failure. On a material level, there is crack formation, delamination, connection breakage and micro-island formation of thin metal layers. These damages are directly detected by microscopic imaging or indirectly by, e.g., measurement of the intensity of IR spectra of the textile polymer or metal content. On an electrical level, capacitance, EMI-shielding and especially electrical conductivity, i.e., electrical resistance, are important and mostly drawn for the indication of functionality and failure.

Considering the electrical resistance, two main types of failure for conductive lines were found. Regarding the course of the electrical resistance during testing, there is sudden fatigue and stepwise fatigue. For sudden fatigue, the resistance/voltage/current signal of the object remains on a constant level during an accelerated ageing experiment until it changes strongly in a fast step. This is caused by the interruption of the conductive line due to the full breakage and was found, e.g., for wires and contact points. The constant remaining signal over a long time period is an advantage of this behaviour. However, on the other side, the remaining lifetime can only be estimated on a statistical basis, not from the measurement of electrical parameters.

For stepwise fatigue, the change of the electrical resistance is a slow but continuous process. It may start with the first cycle of an ageing test and show a certain course with elapsing time or cycle number. The average electrical resistance increases, often with a high slope at the beginning, but then leads into a saturation value. For this behaviour, it needs to be tested, in what way the changing electrical resistance influences a transmitted signal. On the other hand, a fixed course of the electrical resistance provides the possibility of modelling and making the gain of damage predictable just by measuring the electrical resistance. A further and frequent problem of the stepwise fatigue-type failure is an increasing variation of the values, due to a rising gauge factor. The stepwise fatigue does not have a clear point of failure. The end of lifetime needs to be determined ahead in dependence of the resistance level and the gauge factor. Causes for stepwise fatigue are mainly the dissolution of the conductor, delamination of coatings, formation of cracks in coatings and conductive layer breakages.

### 5.4. Cumulative Fatigue Damage Modelling for e-Textiles

Modelling the electrical resistance in dependence of a resistor network for the complex motion of textiles is a theoretical exercise. In the course of this review, an example for modelling the electrical resistance for stepwise fatigue was presented by Xue et al. among stretch ageing [166]. The statistical based lifetime modelling for sudden fatigue-type of failure was shown for bending and stretch ageing by Bossuyt and others [90,103]. Cumulative fatigue damage modelling was performed on different stress levels for e-textiles using the Basquin and Manson–Coffin relations.

These modelling approaches refer to stretching or bending. However, also abrasion, washing, oxidation or chemical attacks impact conductive textiles. Under realistic conditions, even several types of ageing may influence the quality of a textile during its lifetime. To summarize all effects, we propose an extension of the cumulative fatigue damage formula to all ageing categories. A hypothetical formula could sum up all damages of an e-textile during its lifetime. An example following Palmgren, Miner (1) and others could be an “all-in” approach (5):

(5) $$D_{total}=\overset{m}{\underset{i=1}{\sum }}D_{mechanical}+\overset{m}{\underset{i=1}{\sum }}D_{wash}+\overset{m}{\underset{i=1}{\sum }}D_{chemical}+\overset{m}{\underset{i=1}{\sum }}D_{electrical}=1$$

(6) $$\overset{m}{\underset{i=1}{\sum }}D_{mechanical}=\overset{m}{\underset{i=1}{\sum }}D_{stretch}+\overset{m}{\underset{i=1}{\sum }}D_{bend/fold}+\overset{m}{\underset{i=1}{\sum }}D_{abrasion}$$

(7) $$\overset{m}{\underset{i=1}{\sum }}D_{wash}=F\left(T,speed,c_{detergent},t,m\right)$$

(8) $$\overset{m}{\underset{i=1}{\sum }}D_{chemical}=\overset{m}{\underset{i=1}{\sum }}D_{pH}+\overset{m}{\underset{i=1}{\sum }}D_{solvent}+\overset{m}{\underset{i=1}{\sum }}D_{salt}+\overset{m}{\underset{i=1}{\sum }}D_{oxidation}$$

In this proposal, the damage parameter *D_ageing test_* becomes 1, if the performed number of cycles of a treatment reaches the maximum number of cycles to failure, as in the original model. This could be stretching cycles, bending cycles, washing cycles, immersion cycles or immersion time (of maximum immersion time). As the cumulative damage value reaches one the e-textile is not functional any more. The total cumulative damage *D_total_* is a sum of all different kinds of ageing, i.e., mechanical ageing, wash ageing, chemical ageing and electrical ageing. The mechanical damage (6) may be divided into damage from stretching, folding and abrasion. The example of the washing damage shows a function dependent from temperature T, the spinning speed, the concentration of detergent c, washing time t and washing cycles m (7). The chemical damage as damage from different pH values, solvents, salts and oxidation (8).

This summarising formula shows the complexity of impacts on e-textiles, especially when going into detail. It could also be suitable for a simplified description of the performed lifetime investigation and the behaviour of the textile. It is meant to give a more realistic view on the functional ageing of e-textiles because of the multiple impacts.

Considering the functional ageing of e-textiles as an accumulative event, new questions arise and more research is needed, e.g., concerning the interference of ageing methods or damage causes. Are there cross reactions? What role does the sequence of ageing tests play? What role does possible reversible ageing play? These questions need to be answered within each ageing category. However, the influence of the separated ageing mechanisms onto each other and on the overall ageing behaviour of e-textiles needs to be clarified as well.

## 6. Conclusions

The functional ageing of e-textiles is a very complex topic due to the wide variety of material systems and the multiple possible constrains and impacts. On the other hand, this research field is of high importance when considering the rapidly increased applications of e-textile, particularity in the sectors of healthcare and lifeguarding with a very high demand in durability and reliability.

The biggest remaining challenge is the combined character of the ageing behaviour as different mechanisms influence each other and cannot easily be separated for detailed investigations. From the material point of view, fibre chemistry with its specific polymer degradation behaviour and substrate structure (woven, knitted, embroidered) can have influences on the ageing of the conductive functionality. Further, different integration technologies (conductive yarns, TFP, coating and deposition) also show great influence on the functional ageing behaviour, in particular under the mechanical loading of e-textiles. On the other hand, different functional ageing processes (mechanical, chemical and electrical) also often take place simultaneously under typical textile usage conditions. Thus, research on functional ageing of e-textiles requires an interdisciplinarity approach where strong collaboration between different disciplines is required, e.g., material science, textile and polymer chemistry, electrical engineering and testing.

For obtaining a better understanding of the functional ageing behaviour and to predict the usage lifetime and safety of e-textiles, accelerated functional ageing tests are proven to be a suitable tool. Often, it is also utilised to differentiate and highlight new developments of e-textiles. Here, conductivity and electrical resistance, respectively, are the most important and most used as indicators for functional ageing. Some e-textiles also need the electrical capacitance or antenna parameters for characterisation. For the simulation of damages, a wide variety of treatment methods is available in the literature. While typical action modes in mechanical ageing such as stretching, bending and abrasion could be realised separately to investigate their influence in details (Section 3.1), challenges remain in chemical ageing and ageing against washing and laundry. Chemical ageing often combines damages of both conductive structures and textile substrates and mechanical stresses are also involved in washing and laundry; thus, making a separation of different ageing mechanisms difficult (Section 3.2 and Section 3.3).

With regard to the visualisation of damages caused by ageing, scanning electron microscopy, laser microscopy, optical microscopy or X-ray imaging illustrate surficial processes for ageing and non-ageing behaviour. These show two main general mechanisms for the emergence of damage. Firstly, there is the formation of cracks and breakages in conductive layers that lead to a temporary decrease or interruption of electrical currents. There is also the breakage of connections and wires that directly interrupts conductive lines. Secondly, there is the loss of conductive material, which can be due to the delamination of conductive layers, dissolution of conductive material or transformation into a non-conductive material. Hence, also the determination of the metal content is an indicator for functional ageing.

At practical application conditions of e-textiles, it is very often that functional ageing is a combination of all mentioned ageing processes. It remains a big challenge to separately investigate each mechanism independent from each other. Thus, a helpful approach would be the cumulative damage modelling to obtain a combined view on the functional ageing behaviour of e-textiles.

Furthermore, as there are very few standardised ageing testing methods for conductive textiles, e-textiles and smart textiles available, we suggest considering the material system, manufacturing process, mounted devices and planned application to perform lifetime testing using several methods. For wash ageing, the use of the EN ISO 6630 for e-textile testing is quite convincing and appropriate. The same is valid for standardised Martindale abrasion testing according to EN ISO 12947. A similar recommendation is given in [154].

## Data Availability

Not applicable.

## Nomenclature

Accelerated ageing	Experiment in a lab that simulates a possible impact. The expected time period of usage is shortened: often by a cyclic treatment with a higher frequency that in application or with higher temperature or general harsher conditions.
Conductivity	(Here short for electrical conductivity.) The inverse value of the electrical resistance, dimension for quality of a conductor.
Course (knitting)	Direction perpendicular to the direction of knitting.
Electrical resistance	Ratio of voltage and current in an electrical conductor, dimension for quality of a conductor or insulator.
Elongation	Relative extension, mostly in %.
EMI shielding	Protection from electromagnetic interferences, absorption of electromagnetic fields.
Encapsulation	Soft or rigid protection coating.
Galvanostatic charging	A device that keeps a certain current constant in an electrolytic cell.
Gauge factor GF	ΔR/ε; electrical resistance change per elongation.
Non-ageing behaviour	Material behaviour that is not permanent or directly connected with the formation of damage; details in text.
Percolation theory	The randomized occupation of a grid that leads to clusters; here applied for the formation of conductive paths in blends of conductive and non-conductive material.
Ply yarn	Twisted threads that form a yarn.
Voltammetric cycling	Electrical measurement method for recording a current/voltage diagram while varying the voltage.
Wale (knitting)	Direction of knitting.
Warp yarn	Yarn in direction of weaving.
Weft yarn	Yarn perpendicular to direction of weaving.
